# Plasma Cell Leukemia With Spindle-Cell Morphology and CD22 Positivity on Flow Cytometry

**DOI:** 10.7759/cureus.83402

**Published:** 2025-05-03

**Authors:** Agnes I Udoh, Celeste Wagner, Faisal Rawas, Jayati Mallick, FNU Aakash

**Affiliations:** 1 Pathology, University of Texas Medical Branch, Galveston, USA; 2 Hematopathology, University of Texas Medical Branch, Galveston, USA; 3 Hematopathology, Thomas Jefferson University, Philadelphia, USA

**Keywords:** cd22 positive, flow cytometry, multiple myeloma, plasma cell leukemia, sarcomatoid, spindle

## Abstract

Plasma cell leukemia (PCL) is a rare and aggressive form of plasma cell neoplasm, defined by the presence of 5% or more circulating plasma cells in peripheral blood in patients diagnosed with plasma cell myeloma. The spindle-cell morphology in PCL is an exceptionally rare feature, adding diagnostic complexity.

This case involves a 56-year-old female with PCL, showing elongated spindle-shaped plasma cells in the peripheral blood smear. Bone marrow aspirate and core biopsy confirmed the spindle morphology and extensive atypical plasma cell infiltration, with minimal trilineage hematopoiesis. Flow cytometry analysis revealed CD14, CD22, CD27, CD38, CD81, CD138, and cytoplasmic immunoglobulin lambda chain positivity, with absence of CD19, CD20, CD56, CD117, and Kappa light chains.

Spindle cell morphology in plasma cell neoplasms is rare and diagnostically challenging. Additionally, CD22 positivity suggests a unique immunophenotypic profile, potentially representing an intermediate stage between immunoblasts and mature plasma cells. This report documents the first case of CD22-positive PCL with spindle cell morphology.

## Introduction

Plasma cell leukemia (PCL) is a rare, aggressive form of plasma cell neoplasm and is often considered an advanced, circulating phase of multiple myeloma (MM), defined by over 5% plasma cells in the peripheral blood [1-3]. PCL frequently evolves from MM but exhibits unique clinical and molecular features, including frequent CD20 expression and high rates of CD45 negativity. PCL patients typically display more aggressive disease due to high-risk genetic abnormalities and rapid spread beyond the bone marrow. Unlike MM, PCL often presents severe organ damage and poor response to standard therapies, resulting in a significantly shorter survival time. Both PCL and MM originate from abnormal plasma cell proliferation, yet PCL’s distinct immunophenotypic profile and more systemic impact underscore the need for specialized diagnostic and therapeutic approaches.

The bone marrow is usually extensively infiltrated with clonal plasma cells. The immunophenotype differs from other plasma cell neoplasms by its more frequent expression of CD20 and less frequent expression of CD56 [4]. Flow cytometry shows that the neoplastic plasma cells typically express CD38 and CD138. In contrast to normal plasma cells, CD45 is negative or expressed at low levels; CD19 and CD22 are negative, and CD27 and CD81 are frequently negative or under-expressed. Aberrant expressions of antigens are identified in nearly 90% of cases, including CD56, CD200, CD28, KIT, CD20, CD52, CD10, myeloid, and monocytic antigens [2]. Plasma cells are usually round with amphophilic cytoplasm, round eccentric nuclei, perinuclear hof, and coarse chromatin. Morphologic variants of plasma cells in MM are rarely reported [5,6]. We present a unique case of PCL with spindle-cell morphology and CD22 positivity on flow cytometry.

## Case presentation

A 56-year-old female with no significant past medical history presented to the emergency department with persistent shortness of breath, lower chest, and back pain for six weeks. The pain was sharp, constant, and rated as 9/10. A month earlier, she had visited an outside hospital where she was diagnosed with bronchitis and treated with antibiotics and steroids without notable improvement. A week later, she presented at our emergency department with bilateral upper abdominal pain radiating to the flanks, shortness of breath, and night sweats. Initial laboratory evaluation revealed an elevated white blood cell (WBC) count, anemia, and thrombocytopenia, with the presence of plasma cells suggestive of a plasma cell dyscrasia. Imaging identified diffuse liver disease, splenomegaly, and adenopathy. She was referred to hematology and gastroenterology for further evaluation. However, her symptoms worsened, prompting a return to the emergency department. 

**Figure 1:**
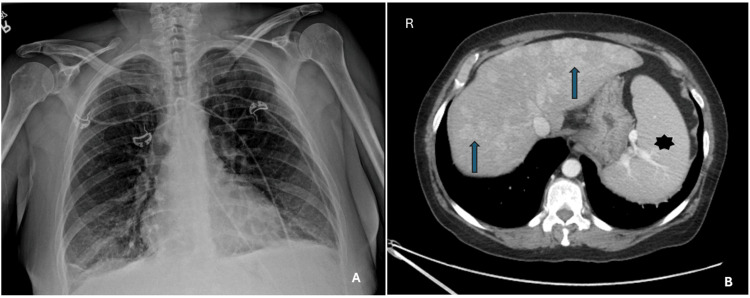
Imaging findings in a 56-year-old female with chest pain, anemia, and thrombocytopenia. (A) Chest x-ray, frontal view, showing hyperexpanded lungs, mild bibasilar linear opacities with no distinct pleural effusion and no pneumothorax. (B) CT scan of the abdomen showing multiple liver nodules (arrows), hepatomegaly, and splenomegaly (star)

On examination, she was tachypneic, tachycardic, wheezing, jaundiced, and pale. A chest x-ray showed hyperexpanded lungs with mild bibasilar linear opacities, no pleural effusion or pneumothorax, and normal cardiac size (Figures 1A, 1B).

An electrocardiogram revealed sinus tachycardia without ST or T-wave changes. Abdominal ultrasound confirmed liver cirrhosis, splenomegaly, and adenopathy. Computed tomography (CT) angiography excluded pulmonary embolism but showed prominent mediastinal lymphadenopathy and noncalcified pulmonary nodules measuring up to 7 mm. A follow-up complete blood count demonstrated marked atypical leukocytosis (30.66 x 10^9^/L), moderate anemia, and thrombocytopenia (Table 1).

**Table 1:** Laboratory findings at current presentation showing anemia, leukocytosis, thrombocytopenia, and metabolic changes. RBC: red blood cell, HPF: high power field, WBC: white blood cell, ALT: alanine transaminase, AST: aspartate aminotransferase, BNP: B-type natriuretic peptide

Labs	Results	Reference range
Complete blood count		
Hemoglobin (g/dL)	6.2	11.6-15
Mean corpuscular volume (fL)	101.7	80.6-95.5
WBCs (10^3^/mcL)	20.66	4.3-11.1
Differential counts (26% atypical plasma cells; absolute leukocytosis with left-shifted neutrophilia, lymphocytosis, and monocytosis)	Abnormal	Normal
Platelets (10^3^/mcL)	39	166-358
Urinalysis		
Appearance	Cloudy	Clear
pH	6.0	4.8-8.0
Blood	2+	Negative
Nitrites	Negative	Negative
RBC/HPF	8	0-3
WBC/HPF	3	0-5
Bilirubin	Negative	Negative
Biochemical parameters		
Blood urea (mg/dL)	19	15-40
Serum creatinine (mg/dL)	0.96	0.6-1.2
Serum sodium (mmol/L)	132	135-145
Serum potassium (mmol/L)	4.2	3.5-5.0
Serum calcium (mg/dL)	11.4	8.6-10.6
Alkaline phosphatase (U/L)	176	34-122
ALT (U/L)	77	5-35
AST (U/L)	68	13-40
Serum cardiac markers		
NT-proBNP (pg/mL)	1,230	<125
Troponin I (ng/mL)	0.005	<0.034

**Figure 2:**
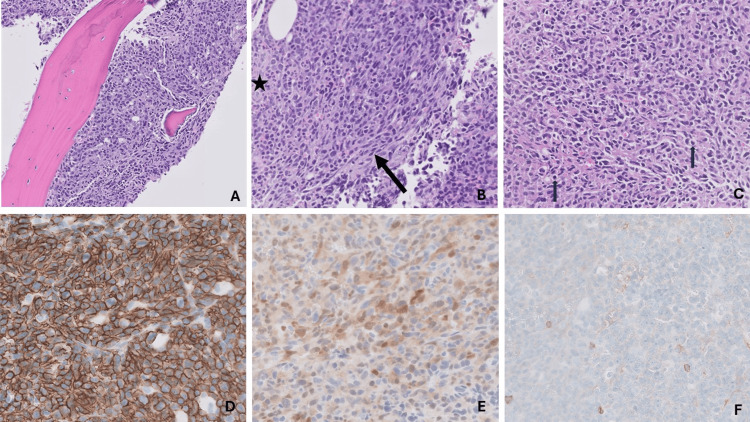
Bone marrow biopsy reveals hypercellularity with infiltration of atypical plasma cells, comprising 90% of bone marrow cellularity. (A) Hypercellular bone marrow. (B) These cells exhibit a spindle shape as highlighted by the black arrow, with minimal remaining normal myelopoiesis indicated by the star (H&E, 10x). (C) Plasma cells with eccentrically placed nuclei and a perinuclear hof indicated by the black arrows. (D) CD138 staining of the membranes of the atypical plasma cells. (E) Cyclin D1 staining of the nucleus of atypical plasma cells. (F) CD56 negative staining.

Serum and urine protein electrophoresis revealed an M spike in the cathodal gamma region of the serum (1.9 g/dL) and a mild increase in gamma globulin. Peripheral blood smear examination showed numerous atypical plasma cells, leading to flow cytometry analysis. Flow cytometry identified an aberrant monoclonal plasma cell population. Bone marrow biopsy revealed hypercellular marrow with a diffuse infiltrate of spindle-shaped plasma cells comprising approximately 90% of marrow cellularity, resulting in markedly decreased trilineage hematopoiesis (Figures 2A-2F).

**Figure 3:**
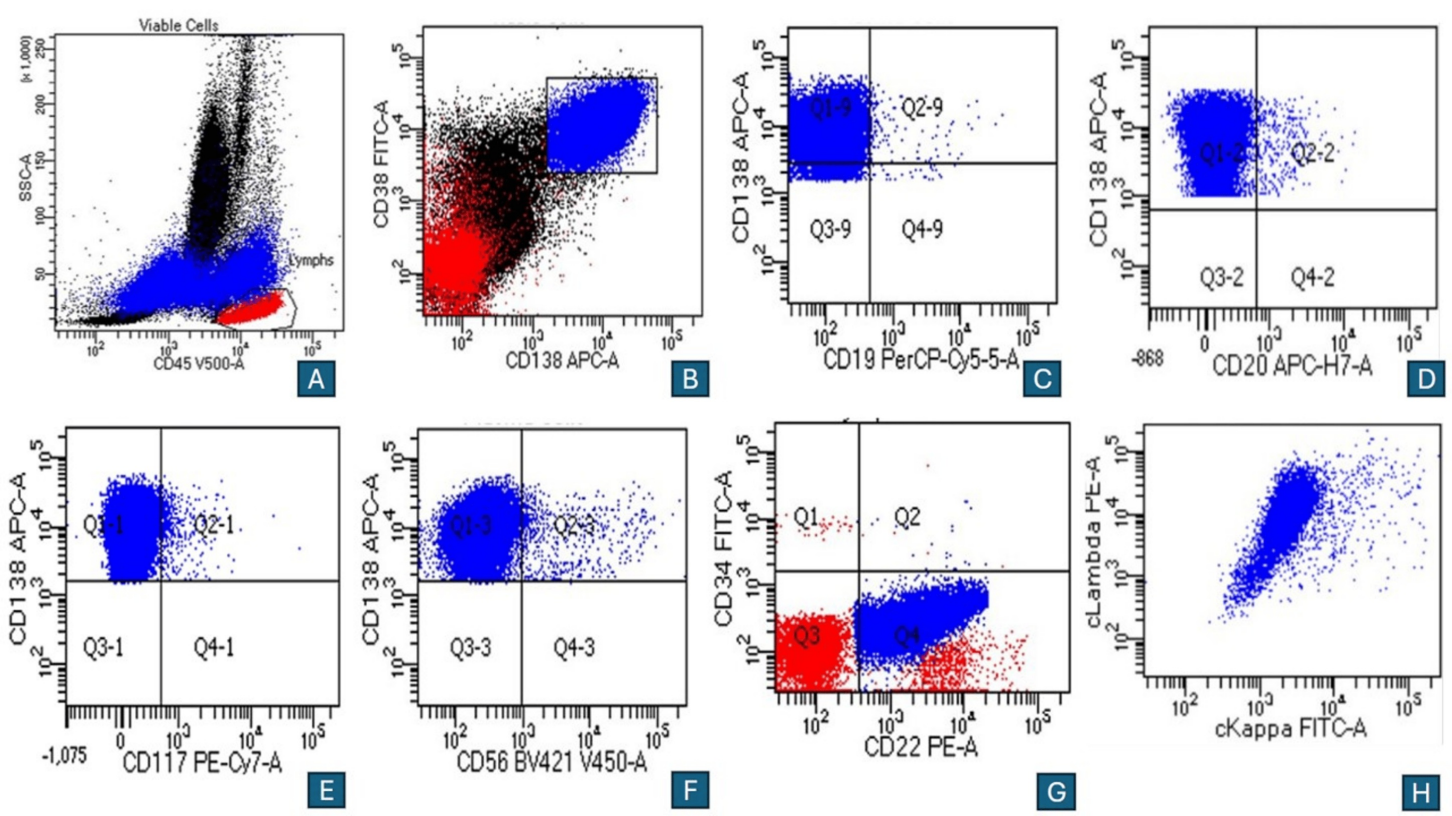
Flow cytometry on bone marrow aspirate shows a bright CD138, CD38, and the neoplastic plasma cell population which is also positive for CD22. Multicolor flow cytometry of bone marrow aspirate reveals an aberrant clonal plasma cell population (blue plots) positive for CD38(B), CD138(B-F), CD22 (G), and negative for CD19 (C), CD20 (D), CD56 (F), CD117 (E), with cytoplasmic lambda light chain restriction (H).

Immunohistochemistry showed plasma cells positive for CD138 and cyclin D1, and negative for CD56. CD117, HHV8, and EBER-ISH were also negative, supporting the diagnosis of a plasma cell neoplasm. Flow cytometry of the bone marrow confirmed similar findings with aberrant plasma cells expressing CD14 and CD22, in addition to CD38, CD138, CD27, and CD81, with kappa light chain restriction (Figures 3A-3H).

Cytogenetic analysis revealed a complex karyotype with loss of chromosomes 13 and 20, and deletions on chromosomes 9q, 14q, and 16q. Fluorescence in situ hybridization (FISH) detected IGH::MAF (t(14;16)) gene rearrangement, monosomy 13, and gains of chromosome 1q, along with chromosomes 5, 9, 11, 15, and 17. Notably, FISH was negative for IGH::CCND1 (t(11;14)), FGFR3::IGH (t(4;14)), and TP53 (17p deletion).

She started chemotherapy. Two days into treatment, she developed suspected community-acquired or hospital-acquired pneumonia and was initiated on cefepime. Despite diuresis and breathing treatments, her shortness of breath worsened, leading to increased work of breathing. She was transitioned to bilevel positive airway pressure (BiPAP) and subsequently transferred to the medical intensive care unit (MICU) for acute hypoxic hypercapnic respiratory failure, eventually requiring mechanical ventilation.

On the fourth day of chemotherapy, she developed tumor lysis syndrome (TLS) with hyperkalemia, necessitating continuous renal replacement therapy (CRRT). She also experienced sustained ventricular tachycardia. Despite aggressive management, her TLS and acute renal failure progressed. After five days in the MICU, she developed acute liver failure and worsening lactic acidosis. Despite maximum vasopressor support, CRRT, and mechanical ventilation, her condition continued to deteriorate. Her prognosis was poor, and she unfortunately passed away two weeks into admission.

## Discussion

This is a unique plasma cell neoplasm clinically presented with lymphadenopathy, leukocytosis, and without any evidence of lytic bony lesions. The morphology and immunophenotype are also distinctive. Spindle cell morphology is rarely seen in plasma cell neoplasm, which could pose diagnostic challenges [7].​​ It demands the exclusion of other spindle cell entities that take place in the bone marrow, such as systemic mastocytosis, metastatic carcinoma, melanoma, sarcoma, or histiocytic/dendritic neoplasms. There are relatively few published cases of plasma cell neoplasms with spindle-shaped/sarcomatous morphology [2,8].​ However, flow cytometric positivity of CD22 was never reported in any previous plasma cell neoplasm. CD22 is a transmembrane glycoprotein expressed by mature B cells, which appears internally during the pro-B-cell stage and becomes expressed on the surface of mature B cells and is lost in the plasma cell phase​ [9,10]. It ​ inhibits signal transduction by the B cell receptor and its co-receptor CD19. CD22 positivity in this plasma cell neoplasm suggests the neoplastic cells are in a stage between immunoblasts and mature plasma cells. This is the first reported case of CD22-positive plasma cell neoplasm with spindle cell/sarcomatoid morphology and presented as PCL.

## Conclusions

This case report highlights an exceptionally rare case of PCL presenting with spindle cell morphology and CD22 positivity on flow cytometry, features not previously documented in plasma cell neoplasms. The atypical spindle morphology adds a unique diagnostic challenge, requiring careful consideration to rule out other spindle cell entities in the bone marrow. Furthermore, the unexpected CD22 expression may suggest a transitional immunophenotypic stage between immunoblasts and mature plasma cells, broadening our understanding of plasma cell differentiation and immunophenotype variability. This case underscores the importance of comprehensive immunophenotypic and morphological analysis in plasma cell neoplasms and may pave the way for further research into the prognostic and therapeutic significance of CD22 in plasma cell disorders. Future studies are needed to investigate the broader implications of CD22 positivity in PCL and its potential role as a prognostic marker or therapeutic target.
